# The Classification of Renal Cancer in 3-Phase CT Images Using a Deep Learning Method

**DOI:** 10.1007/s10278-019-00230-2

**Published:** 2019-05-16

**Authors:** Seokmin Han, Sung Il Hwang, Hak Jong Lee

**Affiliations:** 1 Korea National University of Transportation, Uiwang-si, Gyeonggi-do South Korea; 2 Department of Radiology, Seoul National University Bundang Hospital, Seongnam-si, Gyeonggi-do South Korea; 3 Department of Radiology, Seoul National University College of Medicine, Seoul National University Bundang Hospital, Seongnam-si, Gyeonggi-do South Korea; 4 Department of Nanoconvergence, Seoul National University Graduate School of Convergence Science and Technology, Suwon-si, Gyeonggi-do South Korea

**Keywords:** Deep learning, Renal cancer, Subtype classification, Linear combination

## Abstract

In this research, we exploit an image-based deep learning framework to distinguish three major subtypes of renal cell carcinoma (clear cell, papillary, and chromophobe) using images acquired with computed tomography (CT). A biopsy-proven benchmarking dataset was built from 169 renal cancer cases. In each case, images were acquired at three phases(phase 1, before injection of the contrast agent; phase 2, 1 min after the injection; phase 3, 5 min after the injection). After image acquisition, rectangular ROI (region of interest) in each phase image was marked by radiologists. After cropping the ROIs, a combination weight was multiplied to the three-phase ROI images and the linearly combined images were fed into a deep learning neural network after concatenation. A deep learning neural network was trained to classify the subtypes of renal cell carcinoma, using the drawn ROIs as inputs and the biopsy results as labels. The network showed about 0.85 accuracy, 0.64–0.98 sensitivity, 0.83–0.93 specificity, and 0.9 AUC. The proposed framework which is based on deep learning method and ROIs provided by radiologists showed promising results in renal cell subtype classification. We hope it will help future research on this subject and it can cooperate with radiologists in classifying the subtype of lesion in real clinical situation.

## Introduction

The kidney makes and ejects urine to maintain homeostasis and remove harmful substance. Renal cell carcinoma (RCC) is the most common type of kidney cancer that accounts for 2–3% of human malignancies [1]. According to the cell appearance, RCC can be largely categorized into three subtypes—clear cell renal cell carcinoma (ccRCC), papillary renal cell carcinoma (pRCC), and chromophobe renal cell carcinoma (chRCC). Those three major subtypes constitute more than 90% of the renal cell carcinomas (RCCs) [2]. The ccRCC is known to be the most lethal subtype, whereas the pcRCC and chRCC subtypes have relatively better survival rates [3]. Nowadays, RCC subtype classification is clinically important due to the increased use of novel therapeutic agents, which requires new paradigms to distinguish RCC subtypes [2, 4].

For the subtype classification, visual inspection on computed tomography (CT) images by radiologist is performed to reduce unnecessary biopsy test for subtype classification. There is growing evidence that renal cancer tumor heterogeneity which can be acquired by image scanning can be used in predicting tumor characterization, stage, nuclear grade, response to treatment, and overall survival [2]. To reduce the inspection time and cost, computer-aided diagnosis (CADx) is considered to help radiologists for the medical image interpretation and diagnosis [5, 6]. CADx is applied to differentiate malignancy or benignancy for tumors or lesions [5, 7–9].

Previous researches on the image-based quantitative classification of the renal masses have focused on differentiating benign lesions from malignant ones [10, 11] or malignant ones from normal kidney [12]. Nonetheless, the very next plausible step on this field appears to be studying how to distinguish the malignant subtypes of the renal masses [13, 14], because image features of different RCC subtypes can be used for predicting clinical behavior, treatment response, and overall prognosis [2, 4]. Furthermore, the non-invasive diagnosis might be valuable particularly for elderly patients with small masses [15].

In the previous research about renal cancer subtype classification, Kocaka et al. [13] applied artificial neural network and support vector machine to distinguish the subtypes of renal cancer based on features selected by radiologists. Mileto et al. [14] used dual-energy CT for radiologists to differentiate ccRCC from pRCC visually with iodine mapping.

Our study was designed to develop reproducible and generalizable models for discriminating three major subtypes of RCCs using computed tomography (CT) image analysis along with a machine learning algorithms. To the best knowledge of the authors, this research is the first one about deep learning–based renal cancer subtype classification.

## Methods and Materials

All experimental protocols were approved by Seoul National University Bundang Hospital, Seongnam-si, Kyunggi-do, South Korea. Informed consent was obtained from all patients for their consent to use their information in the research without violating their privacy. A total of 169 renal cancer cases were scanned in Seoul National University Bundang Hospital (Seongnam-si, Kyunggi-do, South Korea). In each case, images were acquired at three phases (phase 1, before injection of the contrast agent; phase 2, 1 min after the injection; phase 3, 5 min after the injection). CT scans were obtained on Philips Brilliance CT 64, IQon, 256 iCT, Siemens Definition edge. The protocol used in image acquisition is presented in Table 1. In Table 1, pitches were 0.641:1 in Philips Brilliance 64, 0.985:1 in Philips IQon CT, 0.993:1 in Philips iCT 256, and 0.6:1 in Siemens Definition edge.

**Table 1:** Image acquisition protocol used in this research

IV contrast	1130cc Xenetix 350 (Guerbet, Aulnay-sous-Bois, France), 3 cc/s
Pre-constrast	Liver dome to ischial tuberosity
50-s delay	Liver dome to ischial tuberosity
5-min delay	Liver dome to genitalia
Pitch	0.641:1–0.993:1
Slice thickness	2.0 mm
kVp/helical rotation	120/0.5 s
Axial reconstruction	5.0 mm standard
Coronal reconstruction	5.0 mm

### Datasets

**Fig. 1:**
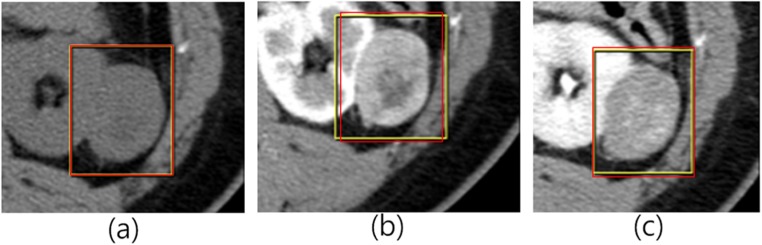
After matching the center of the three-phase images, the ROIs are redrawn. **a** pre-contrast image. **b** 60-s delay phase image. **c** 5-min delay phase image. Yellow rectangle is drawn by radiologists, and red rectangle is redrawn considering ROI of the reference

A total of 169 renal cancer cases were scanned in Seoul National University Bundang Hospital (Seongnam-si, Gyeonggi-do, South Korea). In each case, images were acquired at three phases. There were 57 clear cell cases, 56 papillary cases, and 56 chromophobe cases. We randomly selected 34 test cases (12 chromophobe, 10 papillary, 12 clear). Thus, 135 training cases were used as a training set. After image acquisition, radiologists examined the slices of those three phase images and selected one slice image of renal cancer that seems to be appropriate for diagnosis in each phase. The examination was done in axial view. Thus, we gathered three slice images corresponding to three phases for a case. The biopsy results were used as labels. For a case, radiologists marked the rectangular ROI (region of interest) in each phase image. Each rectangle has a different length and width. Therefore, if we crop the ROI in each phase just as drawn by radiologist, the size of the cropped image may vary. Instead of using the rectangular ROI just as drawn by radiologist, we set the rectangular ROI drawn in the pre-contrast phase as a reference. After matching the center of the ROIs of three phase images considering the reference image, ROIs were redrawn in 60-s delay phase image and 5-min delay phase image to make the width and the height of ROIs equal to that of the reference as shown in Fig. 1.

**Fig. 2:**
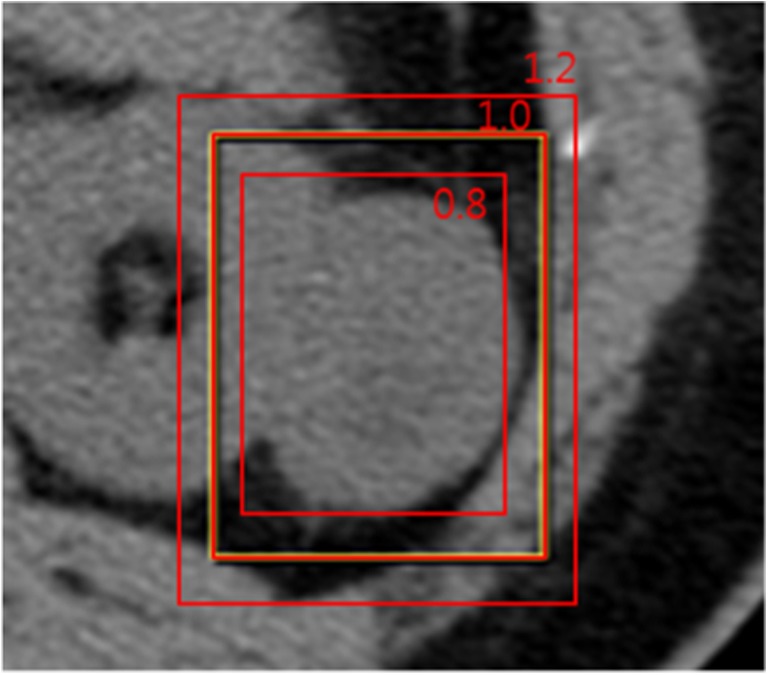
ROI boundaries were rescaled with scale factors 0.8 and 1.2 for image scale augmentation. Original ROI is presented with scale factor 1.0

After redrawing ROIs in each phase image, ROI boundaries were additionally rescaled with a scale factor of 0.8 and 1.2 for image scale augmentation, as illustrated in Fig. 2.

This rescaling process was done in each phase image. Then, images were cropped considering the drawn ROIs. To facilitate the training, the cropped images were resized into patches of 255 × 255 size using bilinear interpolation. Therefore, we made 3 phase × 3 scale images for each patient case. We also employed mirroring augmentation, shuffling of training data, which are provided by Caffe framework [16].

### Network Construction and Training

Rather than using three phase images as they were, we multiplied a combination weight to three phase images. Unfortunately, the optimal combination weight is not known for the three-phase images of renal cancer. Thus, we linearly combined the three channel images to induce the neural network to find the optimal combination weight. This was to improve channel images that had a significant impact on subtype classification.

**Fig. 3:**
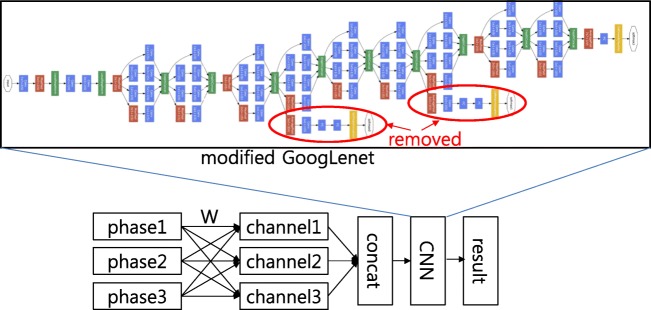
The conceptual architecture of CNN used in this research

This linearly combined three channel images were fed into a CNN (convolutional neural network) after concatenation. For CNN, we employed GoogLeNet [17], which is established in 2014, and modified the network for our purpose. Two auxiliary classifiers were removed in this research. In this research, we assumed two-class problem (for example, chromophobe vs non chromophobe or clear cell vs none clear cell), following the evaluation method of the previous researches [13, 14]. In reference [13], they also performed three-subtype differentiation. However, they reported that three-subtype class differentiation showed relatively poor performance [13]. Because GoogLeNet has originally 1000 class outputs, we modified the network to reduce the output to two class outputs. All pixels in each patch are treated as the input neurons. This is illustrated in Fig. 3.

Because transfer learning followed by fine-tuning is known to show better performance than learning from scratch in many cases [18], the proposed network was initialized by ImageNet [19] pretraining model and was fine-tuned using RCC images. First, a network was trained on other task data, for example, object classification [19]. Then, the trained network parameters were transferred to another network for RCC classification and used as the network initialization. This method is generally used when there are not sufficient data to train complex network with a large amount of parameters. It has been proven that this transfer learning is effective in various domains such as mammography [20], X-ray [21], histology images [22, 23], and retinal images [24, 25]. The input data was three phase images and the label was the corresponding subtype. We used the Caffe [16] deep learning framework and NVidia 1080 GPU to train the CNNs. The network was trained by stochastic gradient descent (SGD) method with base learning rate of 0.0001, learning momentum 0.9, weight decay 0.0002, and a poly learning policy. We used the image batch size of 70 that was the maximum batch size in our system.

## Experimental Results

**Fig. 4:**
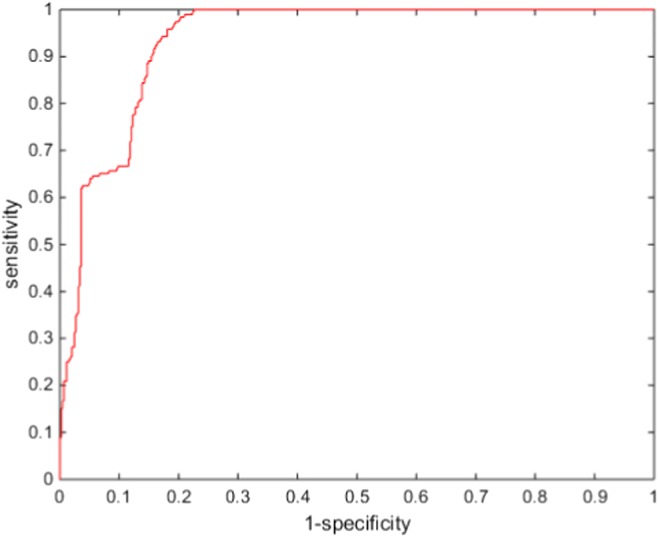
Diagnostic performances of ccRCC classification

**Fig. 5:**
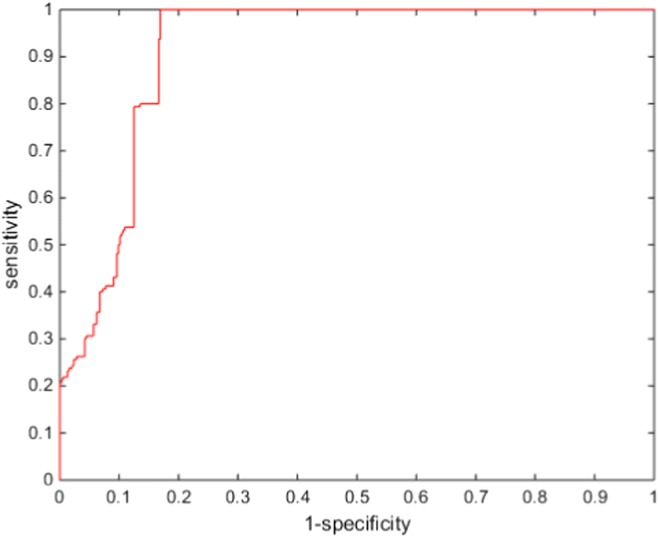
Diagnostic performances of pRCC classification

**Fig. 6:**
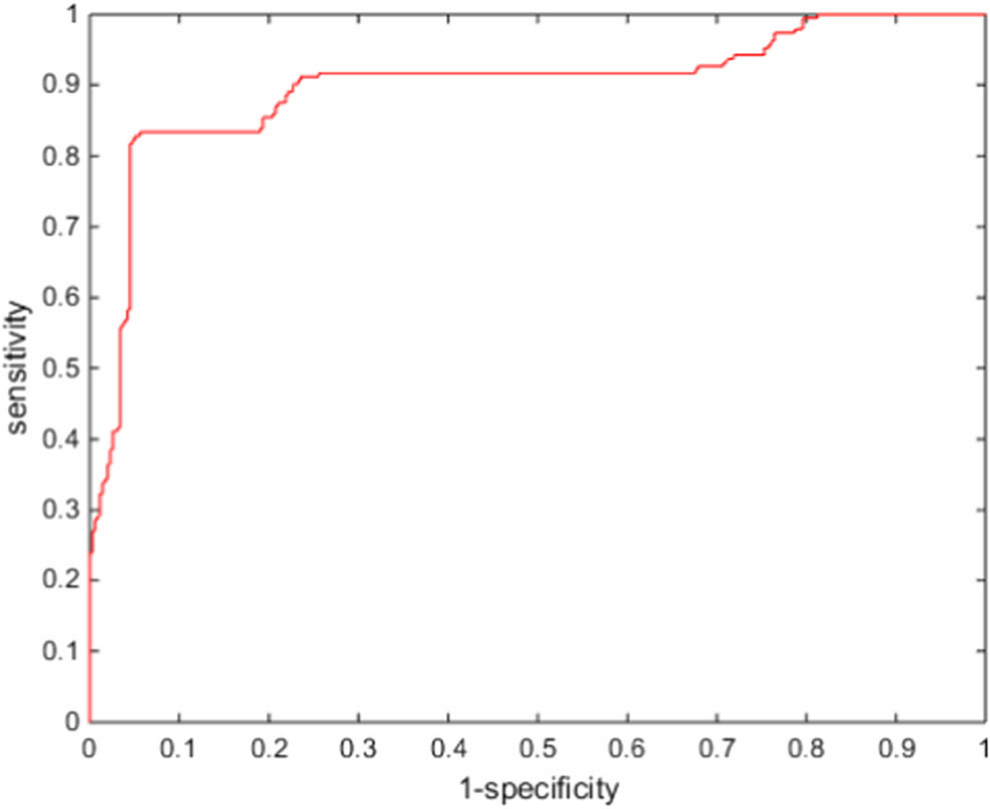
Diagnostic performances of chRCC classification

Because we assumed two-class problem, three experiments were performed (chromophobe vs non-chromophobe, papillary vs non-papillary, and clear cell vs non-clear cell). We presented the performance of the proposed deep learning framework of renal cancer classification in terms of accuracy, sensitivity, specificity, and AUC (area under the curve). Optimal parameters were chosen based on a tenfold cross-validation with the 135 training data. Then, the optimized parameters were applied to evaluate the performance on the 34 test datasets. We presented the performance of ccRCC classification in Fig. 4, pRCC classification in Fig. 5, and chRCC classification in Fig. 6. In the ccRCC classification experiment, chRCC and pRCC were considered as non-clear cell class. In the same way, chRCC and ccRCC were considered as non-papillary renal cell carcinoma class in pRCC classification, and ccRCC and pRCC were considered as non-clear cell class in chRCC classification. We also presented the result summary in Table 2. For three-class problem, which is chRCC vs pRCC vs ccRCC, we presented the result in Table 3, for comparison.

**Table 2:** Diagnostic performances of the proposed CNNs

	Sensitivity	Specificity	Accuracy	AUC
Clear cell	0.6458	0.9353	0.8484	0.9355
Papillary	0.9875	0.8307	0.8694	0.9117
Chromophobe	0.7865	0.9545	0.8879	0.8795

**Table 3:** Diagnostic performances of the proposed CNNs (3 class)

	Sensitivity	Specificity	Accuracy
Clear cell	0.2656	1.0000	0.7335
Papillary	0.4688	0.9609	0.8088
Chromophobe	0.6354	0.9290	0.8241

## Discussion

The proposed method seems to have promising performance in classification of renal cancer. In spite of the fact that the location of the cancers had to be provided by radiologists, the proposed method could accurately classify cancers as chromophobe, papillary, or clear cell. Therefore, the proposed framework can support radiologists to accurately decide the following procedure. For experienced radiologists, the proposed method may not be useful for early detection and diagnosis. However, this method can help those who are not fully trained in radiology.

Though the proposed method seems to work well in the classification of papillary carcinoma and clear cell carcinoma, it did not show good performance in the classification of chromophobe renal cell carcinoma as expected. Because the number of chromophobe cases was close to those of clear cell cases and papillary cases, this may not be due to an imbalance in the ratio of data. It may be because the features obtained from the training data used in this research could not distinguish chromophobe renal cell carcinoma sufficiently from others, while more various features were required to distinguish chromophobe renal cell carcinoma than other renal cell carcinoma subtypes. In that case, we should gather more data to enhance the classification performance.

The motivation to combine each channel linearly was to improve channels that had a significant impact on subtype classification. We applied the learning method to the linear combination weight so that it would be calculated to produce optimum results automatically. Our hypothesis was that the current image adjusted for human inspection may not be the best for the machine learning–based methods. The linear combination of three channel images can be considered as another three channel images which are adjusted for neural network. Considering the result of this research, we can assume that three channel images can be modified and adjusted to have potentially better performance, though their weight cannot be completely fixed. It seems that the best combination weight may vary from task to task. In this research, the best weight calculated by learning algorithm varied from task to task. As mentioned above, those weights for combining each channel linearly were calculated automatically via the learning process itself to be optimized for the training data. If those weights were different from the learned weights, the performance may decrease. In our database, when those weights were set manually without learning process, we guess that the performance may be decreased by 0–8%. This research may give a hint for a potentially new way of inspection with performance improvement. However, validation of the effect of the linear combination of each channel on the performance of human inspector is beyond the scope of this research. To validate the effect, we have to consult radiologists to see whether the combination is more helpful in discriminating the suspicious region than the currently used images. The authors are planning to proceed this in the near future.

For comparison, we referred to the previous research to evaluate the proposed method. In reference [14], radiologists differentiated ccRCC from pRCC visually and showed an AUC of 0.923 (88 cases). In reference [13], machine learning–based approach was used to differentiate ccRCC from non-ccRCC, and it showed an AUC between 0.731 and 0.935 (68 cases). Therefore, we consider that the proposed method shows comparable or better performance. And it should be noted that the proposed method did not use hand-crafted features selected by radiologists, which is the advantage of deep learning method. However, more data is required for a more precise evaluation and generalization.

In evaluation, we refered the method of the previous researches [13, 14] and considered the subtype classification problem as several two-class problems. In reference [13], they reported that three-subtype class differentiation showed relatively poor performance [13]. Their report was in accordance with our results, for we also had relatively poor results in three-subtype class differentiation, as shown in Table 3. That might indicate that there is substantial overlap between subtypes. It also seems to be that while we are given a fixed number of data, the output nodes of three-subtype problem requires more data to be optimized than the output nodes of two-subtype problem does. As mentioned above, we used the GoogLenet modified for our task. At the end of the GoogLenet, softmax function was used for the classification. The number of weights of the softmax function increases as the number of output nodes increases. The number of weights of softmax function in three-subtype problem is 3/2 times larger than the number of weights of softmax function in two-subtype problem. However, more research is required to clarify the reason.

## Conclusion

In this research, we exploit a deep learning framework to differentiate the distinctive subtypes of lesions in renal cancer with CT imaging. A biopsy-proven benchmarking dataset of 169 case images was built and used to evaluate the proposed method. We combined the three-phase input images linearly so that three-phase input images should be reformed and fed into a neural network, which can be also applied in other researches. The networks showed an AUC close to 0.9, regardless of the subtypes. The proposed framework which is based on deep learning method and ROIs provided by radiologists showed promising results in renal cell subtype classification. We hope it will help future research on this subject and it can cooperate with radiologists in classifying the subtype of lesion in real clinical situation.
